# Supplementary material for: Rhodotorula kratochvilovae CCY 20-2-26—The Source of Multifunctional Metabolites
Source: Microorganisms. 2021 Jun 11;9(6):1280. doi: 10.3390/microorganisms9061280 (PMC8231246; doi:10.3390/microorganisms9061280)

microorganisms
MDPI

Suplementary material

Rhodotorula kratochvilovae CCY 20-2-26 – the source of multi-functional metabolites.

Dana Byrtusová [1,2], Martin Szotkowski[2], Klára Kurowska[2], Volha Shapaval[1,] and Ivana Márová[2]

[1] Norwegian University of Life Sciences, Faculty of Science and Technology, Postbox 5003, 1432 Ås, Norway; dana.byrtusova@nmbu.no (D.B.); volha.shapaval@nmbu.no (V.S.)
[2] Brno University of Technology, Faculty of Chemistry, Purkyňova 464/118, 612 00 Brno; xcszotkowski@fch.vut.cz (M.S.); Winklerova10@gmail.com (K.K.); marova@fch.vut.cz (I.M.)
* Correspondence: marova@fch.vut.cz; Tel.: +420-739-997-176

List of Supplementary Tables:

Table S1 *R. kratochvilovae* CCY 20-2-26 growing on various nitrogen sources with glucose as a C-source. Changes of concentration of glucose in the media with individual N-sources [g/L]

Table S2 *R. kratochvilovae* CCY 20-2-26 growing on various carbon sources with urea as N-source. Changes of concentration of individual sugars in the media [g/L].

Table S3 *R. kratochvilovae* CCY 20-2-26 growing on various carbon sources with potassium nitrate as N-source. Changes of concentration of individual sugars in the media [g/L].

Table S4 Total, α- and β-glucan content in *R. kratochvilovae* cells grown in glucose medium with diverse nitrogen sources

Table S5 Total, α- and β-glucan content in *R. kratochvilovae* cells grown at diverse carbon sources with urea as nitrogen source

Table S6 Total, α- and β-glucan content in *R. kratochvilovae* cells grown at diverse carbon sources with potassium nitrate as nitrogen source

List of Supplementary Figures:

Figure S1 Fatty acid composition of lipids produced by *R.kratochvilovae* (glucose medium; different N sources; 96 hour cultivation, C/N 70)

Figure S2 Distribution of carotenoid pigments produced by *R.kratochvilovae* (glucose medium; different N sources; 96 hour cultivation, C/N 70)

Figure S3 Fatty acid composition of lipids produced by *R.kratochvilovae* on different C sources (urea; 96 hour cultivation)

Figure S4 Fatty acid composition of lipids produced by *R.kratochvilovae* on different C sources (KNO3; 96 hour cultivation)

Figure S5 Carotenoid pigment composition produced by *R.kratochvilovae* on different C sources (urea; 96 hour cultivation)

Figure S6 Carotenoid pigment composition produced by *R.kratochvilovae* on different C sources (KNO3; 96 hour cultivation)

Figure S7 Fatty acid composition of lipids produced by *R.kratochvilovae* (mannose medium; different C/N ratio; 96 hour cultivation)

Figure S8 Distribution of carotenoid pigments produced by *R.kratochvilovae* (mannose medium; different C/N ratio; 96 hour cultivation)

Table S1 *R. kratochvilovae* CCY 20-2-26 growing on various nitrogen sources with glucose as a C-source. Changes of concentration of glucose in the media with individual N-sources [g/L]

	Cultivation time						
	1h	24 h	48 h	72 h	96 h	120 h	144 h
KNO_3	29.11±2.75	28.09±2.75	15.07±1.28	9.82±1.74	4.08±0.97	2.31±0.61	1.82±0.74
YE	27.98±1.77	26.98±1.77	16.27±1.58	12.14±2.53	10.63±1.44	6.72±0.87	3.72±0.92
AS	33.01±3.29	31.05±3.29	22.38±1.58	16.13±1.87	14.65±1.10	13.56±0.98	11.27±1.35
NH_4Cl	32.15±2.44	30.08±2.44	20.98±2.08	15.89±2.02	13.62±1.21	11.82±0.97	9.67±1.32
Urea	29.18±2.11	27.68±2.11	18.09±1.27	11.07±2.10	8.80±0.75	3.32±0.81	0.84±0.53

Table S2 *R. kratochvilovae* CCY 20-2-26 growing on various carbon sources with urea as N-source. Changes of concentration of individual sugars in the media [g/L].
.

	Cultivation time [h]						
	1 h	24 h	48 h	72 h	96 h	120 h	144 h
Lactose	34.94±0.48	34.65±0.48	33.98±1.07	34.01±0.87	33.67±0.97	33.07±1.03	32.67±1.24
Mannose	40.42±1.70	34.42±1.70	20.27±1.38	14.14±1.47	12.60±1.04	8.52±0.81	6.72±0.78
Glycerol	37.57±1.29	30.84±1.29	26.48±1.77	19.13±1.65	15.20±0.99	12.64±0.85	11.10±1.15
Xylose	36.75±0.75	29.72±0.75	24.71±1.88	23.68±1.64	20.98±1.02	19.06.±0.97	17.64±1.58
Glucose	40.42±1.85	27.68±2.11	18.09±1.27	11.07±2.10	8.80±0.75	3.32±0.81	0.84±0.53

Table S3 Cultivation on diverse carbon sources with potassium nitrate as N-source. Concentration of individual sugars in the media [g/L]

	Time of cultivation						
	1h	24 h	48 h	72 h	96 h	120 h	144 h
Lactose	35.12±0.42	33.65±0.59	32.98±1.05	33.08±0.80	33.76±0.94	32.97±1.02	32.87±1.13
Mannose	39.85±1.60	23.75±1.35	13.26±1.27	10.11±1.43	6.43±1.14	2.82±0.67	0.62±0.58
Glycerol	35.59±1.39	32.23±1.32	29.38±1.42	25.23±1.27	21.99±1.68	17.82±1.85	14.63±1.05
Xylose	37.08±0.70	33.72±1.38	29.71±1.82	21.28±1.54	16.98±1.27	12.05.±1.24	7.61±1.98
Glucose	39.12±1.45	25.31±1.71	16.55±1.11	11.16±1.10	7.56±0.92	3.61±0.75	1.21±0.11

Table S4 Total, α- and β-glucan content in *R. kratochvilovae* yeast biomass cultivated on different nitrogen sources

N source	Time	48 h	72 h	96 h	144 h
**Pottasium nitrate**	Total glucan	24.22±2.14	22.14±1.54	17.26±1.03	16.55±0.38
	Alpha-glucan	2.16±0.21	3.83±0.23	2.02±0.06	2.16±0.04
	**Beta-glucan**	**22.05±2.21**	**18.31±1.83**	**15.24±1.24**	**14.39±0.77**
**Yeast extract**	Total glucan	27.11±1.54	23.79±1.36	18.46±1.15	14.30±1.03
	Alpha-glucan	2.12±0.13	2.36±0.20	1.76±0.02	1.69±0.01
	**Beta-glucan**	**24.99±1.69**	**21.43±1.57**	**16.70±1.33**	**12.62±1.11**
**Ammonium sulphate**	Total glucan	15.12±0.37	16.59±0.89	17.56±0.67	18.46±1.59
	Alpha-glucan	2.71±0.48	2.62±0.11	1.02±0.04	0.86±0.15
	**Beta-glucan**	**12.41±1.02**	**13.98±1.04**	**16.54±1.00**	**17.60±1.84**
**Urea**	Total glucan	23.45±1.00	22.86±1.05	16.65±0.44	17.94±1.07
	Alpha-glucan	2.51±0.33	2.71±0.21	1.98±0.03	1.01±0.09
	**Beta-glucan**	**20.94±1.25**	**20.15±1.30**	**14.67±0.59**	**16.93±1.13**
**Ammonium chloride**	Total glucan	17.18±0.19	18.69±0.49	15.49±0.16	12.61±0.41
	Alpha-glucan	2.38±0.04	2.98±0.06	2.15±0.14	1.32±0.12
	**Beta-glucan**	**14.80±0.81**	**15.71±0.66**	**13.33±0.85**	**11.30±0.75**

Table S5 Total, α- and β-glucan content in *R. kratochvilovae* cells grown at diverse carbon sources with urea as nitrogen source

C source	Time	48 h	72 h	96 h	144 h
**Lactose**	Total glucan				
	Alpha-glucan	-	-	-	-
	**Beta-glucan**				
**Mannose**	Total glucan	17.96±0.68	11.83±0.43	11.12±1.57	10.54±0.49
	Alpha-glucan	4.21±0.12	1.23±0.02	0.84±0.11	0.67±0.14
	**Beta-glucan**	**13.75±0.99**	**10.59±0.76**	**10.28±1.83**	**9.87±0.67**
**Glycerol**	Total glucan	5.64±0.22	5.19±0.33	5.28±0.24	6.31±0.60
	Alpha-glucan	1.82±0.07	1.00±0.04	0.28±0.02	0.95±0.21
	**Beta-glucan**	**3.83±0.54**	**4.19±0.67**	**5.00±0.37**	**5.37±0.80**
**Xylose**	Total glucan	4.56±0.16	9.88±1.02	8.87±0.76	8.75±0.55
	Alpha-glucan	1.60±0.01	1.60±0.09	1.28±0.04	1.71±0.13
	**Beta-glucan**	**2.96±0.39**	**8.28±1.13**	**7.59±0.91**	**7.04±0.76**

Table S6 Total, α- and β-glucan content in *R. kratochvilovae* cells grown at diverse carbon sources with potassium nitrate as nitrogen source

C source	Time	48 h	72 h	96 h	144 h
**Lactose**	Total glucan				
	Alpha-glucan	-	-	-	-
	**Beta-glucan**				
**Mannose**	Total glucan	18.86±1.08	17.43±1.30	20.70±0.29	21.53±1.46
	Alpha-glucan	1.73±0.13	1.81±0.06	2.57±0.42	2.07±0.13
	**Beta-glucan**	**17.13±1.24**	**15.62±1.67**	**18.14±0.58**	**19.46±1.85**

**Glycerol**	Total glucan	8.64±0.36	7.17±0.15	5.39±0.41	7.24±0.20
	Alpha-glucan	2.03±0.08	2.56±0.10	2.31±0.06	4.55±0.08
	**Beta-glucan**	**6.61±0.49**	**5.36±0.41**	**3.08±0.67**	**2.69±0.34**
**Xylose**	Total glucan	16.57±0.99	16.43±0.44	15.23±1.00	13.80±1.05
	Alpha-glucan	2.49±0.21	2.85±0.13	3.38±0.32	3.80±0.04
	**Beta-glucan**	**14.08±1.05**	**13.58±0.81**	**11.85±1.45**	**10.00±1.27**

Figure S1 Fatty acid composition of lipids produced by *R.kratochvilovae* (glucose medium; different N sources; 96 hour cultivation, C/N 70)

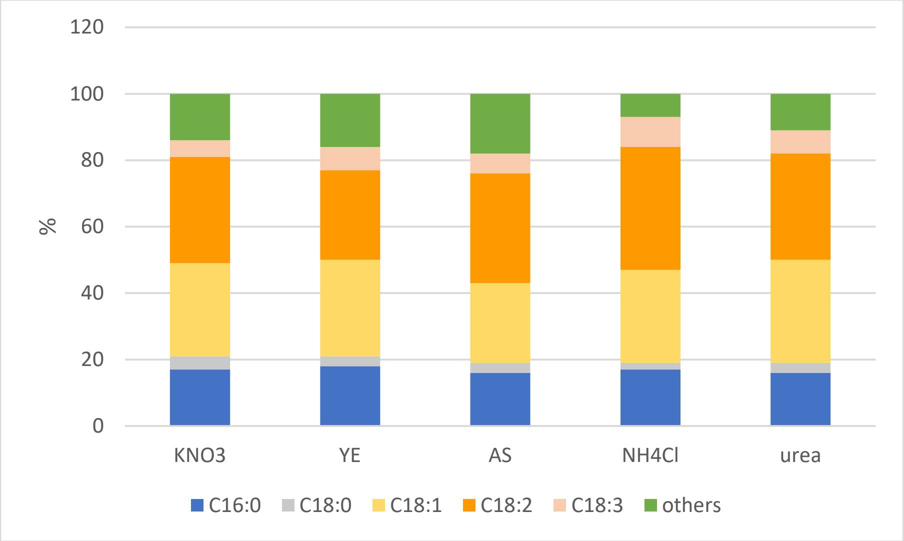

Figure S2 Distribution of carotenoid pigments produced by *R.kratochvilovae* (glucose medium; different N sources; 96 hour cultivation, C/N 70)

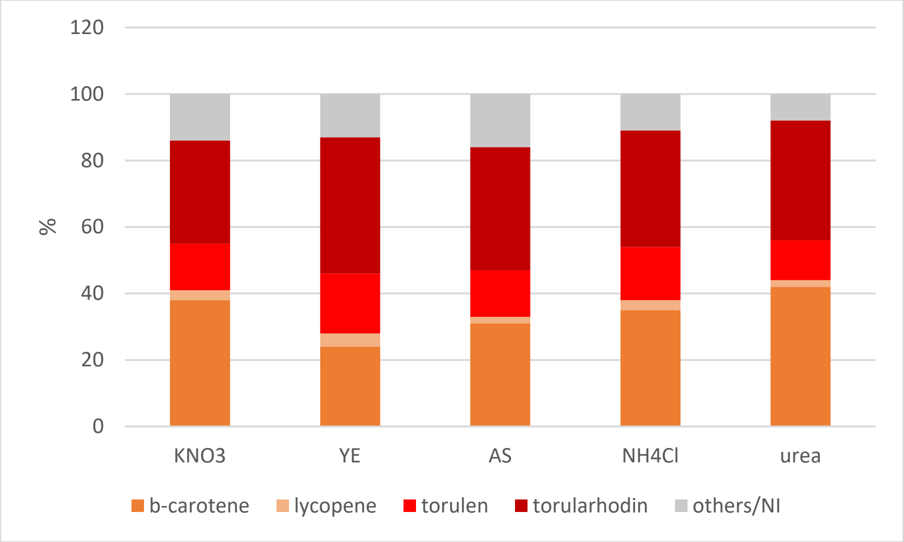

Figure S3 Fatty acid composition of lipids produced by *R.kratochvilovae* on different C sources (urea; 96 hour cultivation)

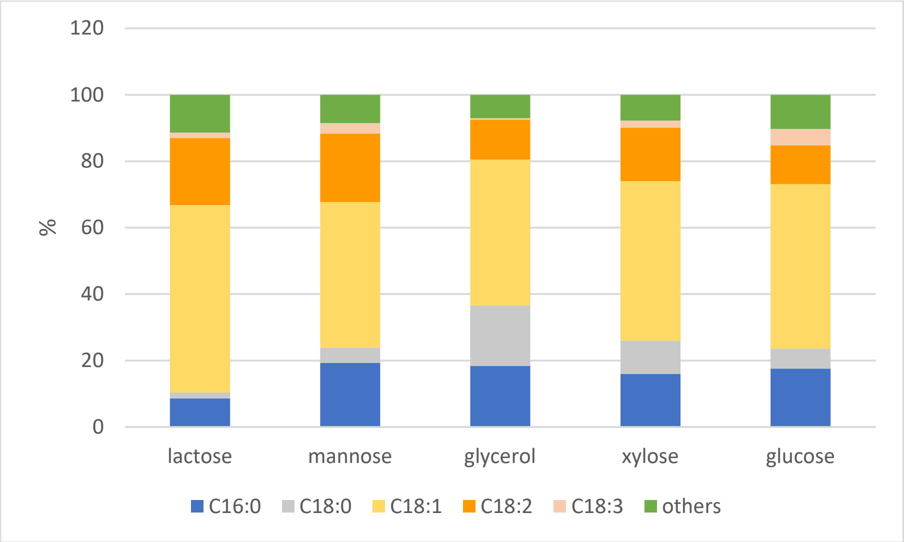

Figure S4 Fatty acid composition of lipids produced by *R.kratochvilovae* on different C sources (KNO3; 96 hour cultivation)

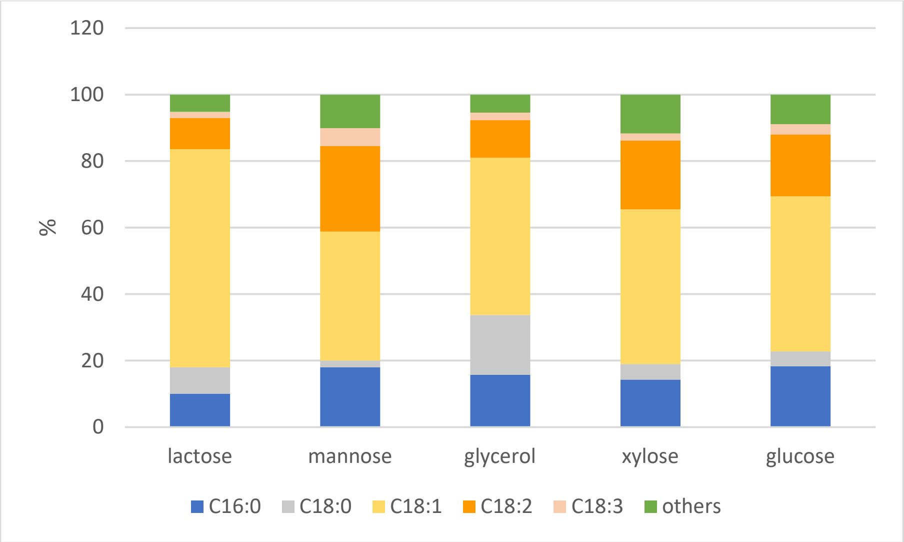

Figure S5 Carotenoid pigment composition produced by *R.kratochvilovae* (glucose medium; urea; 96 hour cultivation)

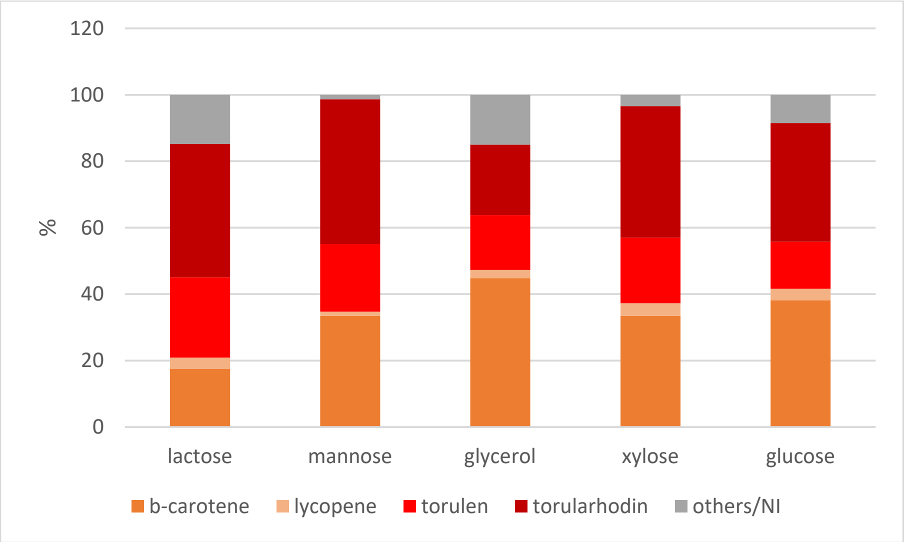

Figure S6 Carotenoid pigment composition produced by *R.kratochvilovae* (glucose medium; KNO3; 96 hour cultivation)

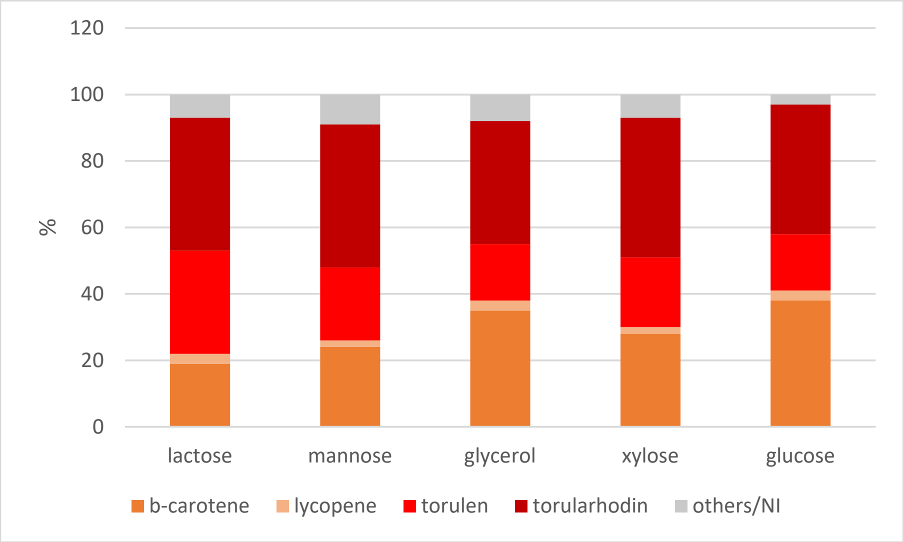

Figure S7 Fatty acid composition of lipids produced by *R.kratochvilovae* (mannose medium; different C/N ratio; 96 hour cultivation)

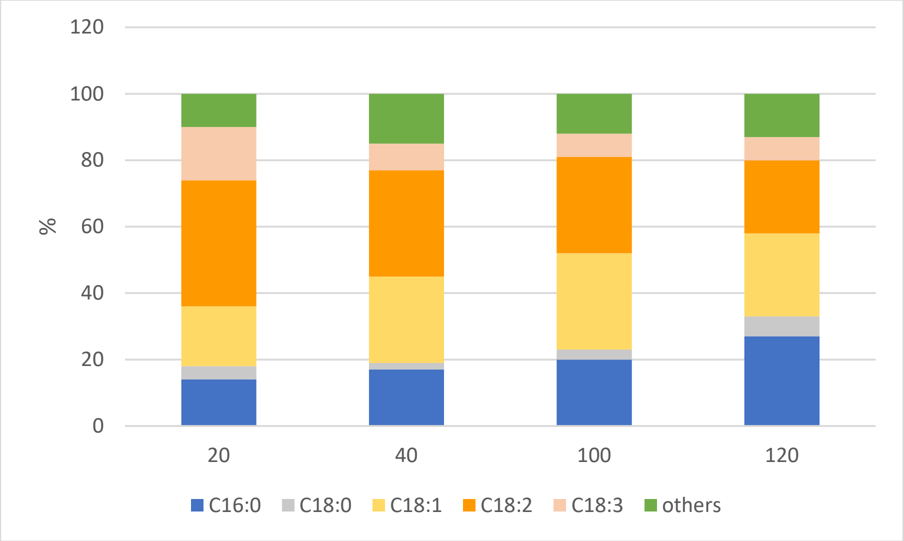

Figure S8 Distribution of carotenoid pigments produced by *R.kratochvilovae* (mannose medium; different C/N ratio; 96 hour cultivation)

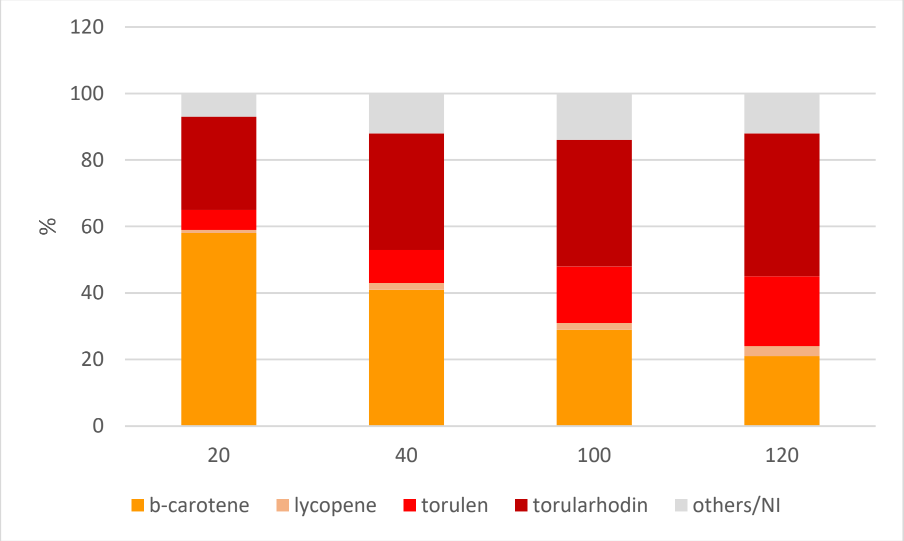

Supplement: Supplementary file 1 [file microorganisms-09-01280-s001.zip › microorganisms-1223003-supplementary.pdf]
